# Supplementary material for: Cytosolic Phospholipase A2 Is Required for Fexofenadine’s Therapeutic Effects against Inflammatory Bowel Disease in Mice
Source: Int J Mol Sci. 2021 Oct 15;22(20):11155. doi: 10.3390/ijms222011155 (PMC8539349; doi:10.3390/ijms222011155)
Supplement: Supplementary file 1 [file ijms-22-11155-s001.zip › ijms-1414967-supplementary.pdf]

Cytosolic Phospholipase A2 is Required for Fexofenadine's Therapeutic Effects Against Inflammatory Bowel Disease in Mice

Xiangli Zhao[1], Ronghan Liu[1], Yuehong Chen[1], Aubryanna Hettinghouse[1],

Chuan-ju Liu[1,2,*]

Supplementary Table S1. Scoring of clinical symptoms. Clinical symptoms include rectal bleeding, body weight loss, and stool consistency.

Score of Clinical indicators	symptoms
Rectal Bleeding	
0	negative
2	blood trace
4	gross blood
Body weight loss	
0	less than 1%
1	between 5% and 10%
2	between 10% and 15%
3	between 15% and 20%
4	over 20%
Stool consistency	
0	normal
2	loose stool
4	diarrhea

Supplementary Table S2. The comparison of the clinical symptoms among H1R KO mice, cPLA2 KO mice, and corresponding WT mice at experimental endpoint. DSS-induced colitis model was established in 8-week-old indicated mice with ad libitum access to with drinking water containing 3% DSS for 10 days, followed by normal drinking water for 3 days, and then sacrificed. Data are mean ± SE; * $p<0.05$.

	**WT**	**H1R KO**	**significance**
Bleeding score	2.8	2.5	ns
Stool score	2.7	2.4	ns
Body weight score	0.78	0.81	ns
colon length	5.2 cm	4.8 cm	ns

	WT	cPLA2 KO	significance
Bleeding score	2	1.6	*
Stool score	1.7	1.9	ns
Body weight score	0.85	0.95	ns
colon length	4 cm	4 cm	ns

The mean value of the clinical scores at endpoint day
